# Maternal allergen immunisation to prevent sensitisation in offspring: Th2-polarising adjuvants are more efficient than a Th1-polarising adjuvant in mice

**DOI:** 10.1186/1471-2172-11-8

**Published:** 2010-03-01

**Authors:** Linda K Ellertsen, Unni C Nygaard, Ingrid Melkild, Martinus Løvik

**Affiliations:** 1 Department of Environmental Immunology, Division of Environmental Medicine, Norwegian Institute of Public Health, Oslo, Norway; 2 The Gade Institute, Section for Microbiology and Immunology, University of Bergen, Bergen, Norway; 3 Insitute of Cancer Research and Molecular Medicine, Norwegian University for Science and Technology, Trondheim, Norway

## Abstract

**Background:**

Allergy has been an increasing problem in several parts of the world. Prenatal exposure to allergen and microbial components may affect the development of allergies in childhood, as indicated by epidemiological and experimental studies. We investigated the capacity for allergic sensitisation in offspring after induction of a Th1- or a Th2-polarised immune response to the same allergen in mothers during pregnancy.

**Results:**

During pregnancy, mice were immunised with ovalbumin (OVA) given with either one of the Th2-adjuvants pertussis toxin (PT) or Al(OH)_3 _(aluminium hydroxide), or with the Th1 adjuvant CpG. Offspring were immunised with OVA in Al(OH)_3 _as young adults. Serum and supernatants from *ex vivo *stimulated or non-stimulated spleen cells from mothers and offspring were analysed for OVA-specific antibodies and cytokines, respectively. Mothers immunised with OVA together with either Al(OH)_3 _or PT had increased levels of OVA-specific IgE and IgG1 compared to naive mothers, whereas mothers immunised with OVA together with CpG had increased levels of OVA-specific IgG2a compared to naive mothers. In general the highest levels of IL-5, IL-10, and IFNγ were observed in spleen cells from mothers immunised with PT and OVA. Upon immunisation, offspring from mothers immunised with OVA and either PT or Al(OH)_3 _showed reduced levels of OVA-specific IgE and IgG1 and increased levels of OVA-specific IgG2a antibodies compared to offspring from naive mothers. Maternal immunisation with CpG and OVA did not affect antibody responses in offspring.

**Conclusion:**

Allergic sensitisation in the offspring was affected by the type of adjuvant used for immunisation of the mothers with the same allergen. Th2 polarisation of the immune response in the mothers was found to give reduced IgE levels upon sensitisation of the offspring, whereas no reduction was achieved with Th1 polarisation in the mothers.

## Background

The prevalence of allergy has been increasing in westernised countries, and allergic diseases represent a major burden for the patients and the society. Together with early childhood, the gestational period appears to be important in relation to the immune system and the development of allergy [1,2]. Allergen-specific immune responses in cord blood mononuclear cells (CBMCs) have been detected already at 22 weeks of gestation [1]. Reduced mitogen- and allergen-induced IFNγ secretion in CBMCs has been reported in children who subsequently developed allergy [3,4]. These findings suggest foetal allergen priming. However, the responses observed may be non-specific rather than an allergen-specific [5]. Increased total cord blood IgE levels has been reported in children who develop allergy later in life [6,7]. If the immune system can be primed *in utero *for development of allergy, prevention of allergic disease should start before birth.

Previously, our group has found reduced allergic sensitisation in mouse offspring after immunisation of mothers during pregnancy with allergen together with the adjuvant Al(OH)_3 _(inducing predominantly a Th2- type of immune response) [8]. A cross-regulation between Th1 and Th2 cells, resulting in reciprocal inhibition has been suggested as a cause for the dominance of either a Th1- or a Th2 response to an antigen in an individual. Allergy is primarily associated with a Th2-type of immune response, while Th1-promoting factors have been proposed to reduce the risk for developing allergy [9]. In the mother-offspring mouse model, we wanted to study if polarisation of the maternal immune response towards a Th1 or a Th2 immune response using microbial components as adjuvants would differently influence sensitisation in offspring. Mothers were immunised with OVA given with either PT (Th2 adjuvant) or CpG (Th1 adjuvant) during pregnancy. Mothers immunised with the Th2-adjuvant Al(OH)_3 _and OVA used in previous studies served as positive controls. Sensitisation was studied in offspring after immunisation with OVA and Al(OH)_3 _at 6 weeks and OVA alone at 8 weeks of age. Sera from mothers and offspring were analysed for OVA-specific antibodies and spleen cells were analysed for cytokine release (IL-5, IL-10 and IFNγ). The findings challenge common perceptions regarding the role of Th1- and Th2-promoting environmental factors during pregnancy in relation to allergy development.

## Methods

### Mice

Female and male inbred NIH/OlaHsd mice (age 6 to 7 weeks at arrival from Harlan UK Ltd. (Oxon, England)) were housed on BeeKay bedding (B&K Universal AS, Nittedal, Norway). NIH/OlaHsd mice have good breeding properties, and are good antibody responders with a mixed Th1-Th2 immune response. The mice were housed in type III macrolon cages in Thorens maximiser racks with standard Hepa filter (Thoren Caging system, Hazleton, Pennsylvania, USA), females and males on separate sides of the rack. The mice were exposed to a 12 h/12 h light/dark cycle at room temperature (20 ± 2 °C), and 40-60% relative humidity. Female mice were given pelleted food RM3 for extra nutrition during pregnancy, while males and offspring were given RM1 from SDS (Essex, England) and tap water *ad libitum*. To start the oestrus cycle, female mice were given paper and bedding from the males' cages on days 15, 16 and 17 after arrival [10]. On day 18, one male was placed in each cage with three females. The next three days the female mice were checked for vaginal plugs, and plugged mice were moved to separate cages. If the plugged female turned out to be pregnant, the first day of pregnancy was considered to be the day after the plug was observed.

The experiments were performed in conformity with the laws and regulations for experiments with live animals in Norway, and they were approved by the Experimental Animal Board under the Ministry of Agriculture in Norway.

### Reagents used in the immunisation protocol

For all immunisations ovalbumin ((OVA), chicken egg albumin, Grade VII, Sigma, St Louis, USA) was dissolved in Hanks Balanced Salt Solution (HBSS, Gibco BRL, Paisly, UK). When given with an adjuvant, the dissolved OVA was mixed with either aluminium hydroxide gel adjuvant (3% Al(OH)_3_, Brenntag Biosector, Fredriksund, Denmark), pertussis toxin (bordetella pertussis, CalBiochem, Cat. no 516560, Nottingham, England) or CpG 1826 ('5-TCC ATG ACG TTC CTG ACG TT-2'; TIB Molbiol, Berlin, Germany). The doses used for the different reagents are given in Table 1.

**Table 1:** Immunisation protocol and time points for collection of blood and spleens.

Host	Time-points	Maternal treatment			
		
		PT mothers	CpG mothers	Al(OH)_3 _mothers	Naive mothers
**Mothers**	**Day 3 of pregnancy.**	Blood samples (T0)			
		
		10 μg OVA + 0.5 μg PT N = 10	10 μg OVA + 50 μg CpG N = 10	10 μg OVA + 2 mg Al(OH)_3_ N = 10	Not immunised N = 10
	
	**Day 10 of pregnancy**	1 μg OVA	1 μg OVA + 5 μg CpG	1 μg OVA + 0.2 mg Al(OH)_3_	Not immunised
	
	**Day 10 after delivery**	Blood samples (T10)			
	
	**3 weeks after delivery**	Exsanguinated (TT): serum and spleens collected			

**Offspring**	**6 weeks**	Blood samples (T0) Immunised: 10 μg OVA + 2 mg Al(OH)_3_ N = 40 offspring per maternal treatment group			
	
	**8 weeks**	10 μg OVA			

	**9 weeks**	Exsanguinated (TT): serum and spleen cells were collected			

### Experimental protocol for mothers

Plugged mice were randomised to groups to be given Al(OH)_3_, PT, CpG, or to be naive mothers (10 mothers per treatment group). The adjuvants were given mixed with OVA (see Table 1 for immunisation protocol). During pregnancy mice were immunised by subcutaneous injection (0.2 ml) on days 3 and 10 of pregnancy (Table 1). Before immunisation on day 3 of pregnancy (T0) and on day 10 after giving birth (T10), the mice were bled from the lateral femoral vein into heparinised capillary tubes after puncture of the vessel with a 21-G needle [11]. After weaning of the offspring (3 weeks after birth), blood was collected from the dams by heart puncture under CO_2 _anaesthesia and the spleens were excised.

### Experimental protocol for offspring

Three weeks old, the offspring were weaned from the mothers, marked by ear punching and separated by gender. All offspring were bled at 6 weeks of age (T0), and immunised intraperitoneally (0.2 ml) at 6 and 8 weeks (Table 1). Two males and 2 females per mother (n = 160, 40 mice per category of mother) received OVA with Al(OH)_3 _at 6 weeks and OVA alone at 8 weeks. Nine weeks old (TT) the offspring were anaesthetised with CO_2 _and bled by heart puncture. Spleens were collected from 48 of the immunised offspring.

### ELISA for detection of OVA specific IgE, IgG2a, and IgG1

The detection of OVA-specific IgE has previously been described by Løvik *et al. *[12] and modified by Ormstad *et al. *[13]. OVA- specific IgG2a- and IgG1 antibodies were determined as described by Nygaard *et al. *[14,15]. However, the avidin-biotin-complex (ABC) - alkaline phosphatase (ALP) reagent from Dako became unavailable during the period of serum analyses. Therefore, a new procedure for colour development in the IgE and IgG2a assays was introduced. Poly-horseradish peroxidase (HRP) streptavidin (Thermo Scientific, Cat. No N200, Diagen AS) was diluted 1:40 000 in 4% BSA/PBS and 100 μl was added to each well. After incubation for 1 hour at 21°C, the plates were washed and 100 μl of TBM Stabilized Chromogen (Cat. No SB02, Biosource, Invitrogen, Carlsbad, CA, USA) solution was added to each well and incubated for 15 minutes at room temperature. Thereafter 2 N H_2_SO_4 _(50 μl/well) was added to stop the colour development process. The serum used for standard curve generation in the modified IgE assay was replaced by mouse anti-OVA IgE from Serotec (MCA 2259). The absorbance (OD) was measured at 405 nm with the original colour development (IgE and IgG2a) and 450 nm with the new colour development (IgE, IgG2a, and IgG1) on a MRX Microplate Reader (Dynatech laboratories, Chantilly, VA, USA) connected to a PC using BioLinx software (Dynatech Laboratories) for instrument operation and calculations. The antibody levels are expressed in arbitrary units (AU)/ml, which was calculated from standard serum pool for IgG1 and IgG2a, whereas IgE was defined by the standard mouse anti-OVA IgE from Serotec. The modified IgG2a and IgE assays were used for analysis of sera from mothers at time-points T0 and T10, while in the offspring the modified IgG2a assay was used at time-point T0. For all other analyses the original assays were used. The quantitative ranges for the IgE, IgG1, and IgG2a assays are about 2, 3, and 4 logs, respectively. All samples at each given time-point were analysed on the same day and with the same version of the ELISA assay. The average coefficients of variation between ELISA plates analysed on the same day for IgE, IgG1, and IgG2a were 7%, 6%, and 13%, respectively. If samples were below or above the quantitative detection limits of the assay, they were given a fixed value just below or just above the quantitative detection limits, respectively.

### Ex vivo quantification of spleen cell cytokine production

The spleen was removed and a single cell suspension prepared as described previously by Vinje *et al. *[16]. The spleen cells were seeded on 24-well culture plates (Coaster 3473, Incorporated, NY, USA) to a final concentration of 5 × 10^6 ^cells/ml. OVA was added to a final concentration of 1 mg/ml OVA. The cells were incubated at 37°C and 5% CO_2 _for 72 hours or 6 days in medium alone, or with OVA for 6 days. Thereafter the plates were centrifuged, and supernatants were collected and stored at -80°C. The concentrations of IL-5, IFNγ, and IL-10 in cell culture supernatants were determined by ELISA according to the protocols provided by the manufacturer (Mouse Duo Set, R&D Systems, Inc, Minneapolis, USA). The culture supernatants were added to duplicate wells and analysed undiluted, diluted 1:10, or 1:100 depending on the expected concentration of the cytokines. The absorbance was measured at 450 nm (with a reference filter for correction at 550 nm), as described above.

### Data analysis

For statistical analysis of responses in offspring, the mothers were used as the experimental unit, since the offspring from each mother are not independent. This means that the average value from all the offspring from each mother was used. A two-sided Kruskal Wallis test with Dunn's post hoc test versus one control group was used for all comparisons. All analyses were performed with GraphPad Prism 5.0. In the text and tables, group medians are given with 25- and 75- percentiles in parentheses. P < 0.05 was considered statistical significant.

## Results

### Antibody and cytokine responses in mothers immunized with OVA and PT, CpG, or Al(OH)_3_

**Figure 1:**
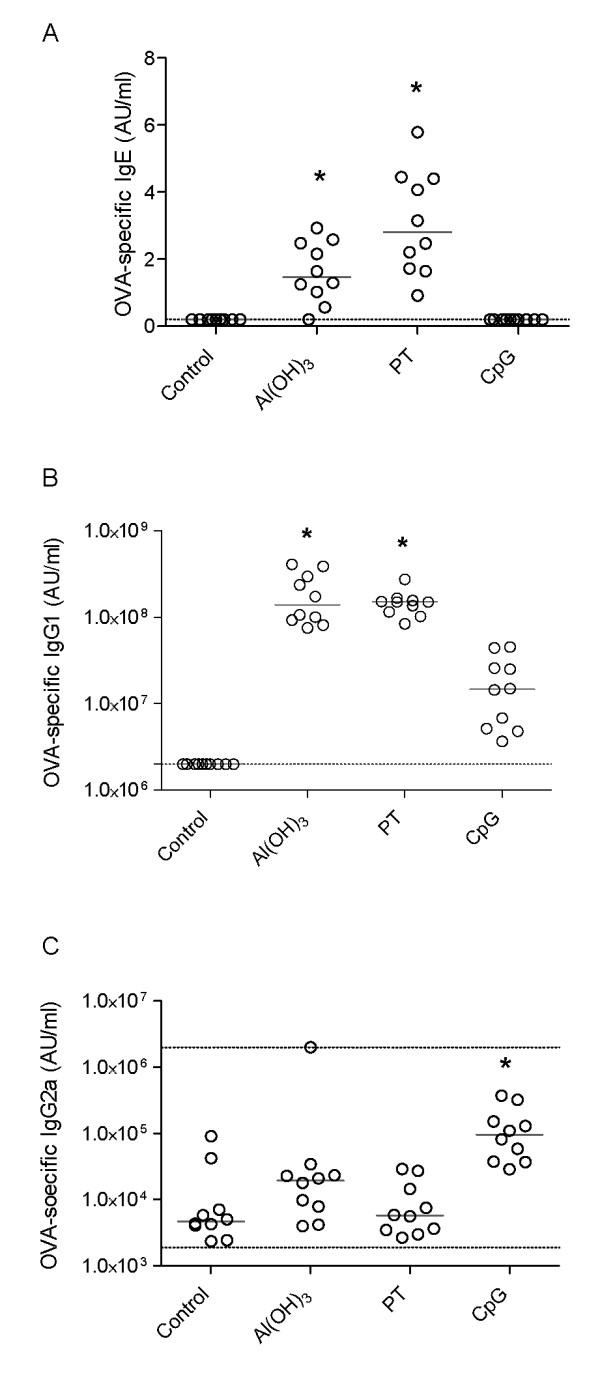
**Antibody responses in mothers at weaning**. Mothers were immunised with the allergen ovalbumin (OVA) together with either Al(OH)_3_, PT, or CpG. Naïve mothers served as negative controls. OVA-specific IgE (A) and IgG1 (B), and IgG2a (C) antibodies were measured at weaning and the antibody concentrations are measured in arbitrary units (AU/ml). The median values are indicated by a line, * denotes a statistically significant difference compared to the OVA group, p < 0.05.

First, we examined whether the adjuvants used had induced a Th1- or Th2-dominant immune response in the mothers as intended. Blood samples were taken from the mothers on day 3 of pregnancy (T0) to determine antibody levels before immunisation, at 10 days after delivery (T10), and at the end of the lactation period (TT) (Table 1). As expected, no OVA-specific antibodies were detected before pregnancy (data not shown). At weaning (TT), significantly higher levels of anti-OVA IgE and IgG1 were observed in Al(OH)_3_- and PT- treated mothers as compared to naive mothers (Figures 1A and 1B). In contrast, CpG immunised mothers had significantly higher levels of anti-OVA IgG2a antibodies but not IgE and IgG1 antibodies compared to naive mothers (Figure 1C). A similar antibody-pattern was seen at 10 days after delivery (Table 2).

**Table 2:** OVA-specific antibody levels in mothers 10 days after delivery (during lactation period).

	Al(OH)_3 _mothers	PT Mothers	CpG mothers	Naive mothers
**IgE**	2150 (1673-2360)*	4094 (2261-4953)*	90 (90-90)	90 (90-90)

**IgG1**	2.5 × 10^8^ (1.6 × 10^8^-4.2 × 10^8^)*	3.0 × 10^8^ (2.4 × 10^8^-4.4 × 10^8^)*	4.2 × 10^7^ (2.1 × 10^7^-7.8 × 10^7^)	2.0 × 10^6^ (2.0 × 10^6^-2.1 × 10^6^)

**IgG2a**	1.9 × 10^5^ (9.9 × 10^4^-3.3 × 10^5^)	2.1 × 10^5^ (9.6 × 10^4^-10 × 10^5^)	1.5 × 10^6^ (9.6 × 10^5^-5.2 × 10^6^)	2.6 × 10^5^ (1.1 × 10^5^-1.2 × 10^6^)

Mothers were immunised during pregnancy with either OVA + Al(OH)_3_, OVA + *pertussis toxin *(PT), OVA + CpG, or were not unimmunised. OVA-specific antibody levels are given as medians with 25- and 75-percentiles (AU/ml). * p < 0.05.

**Figure 2:**
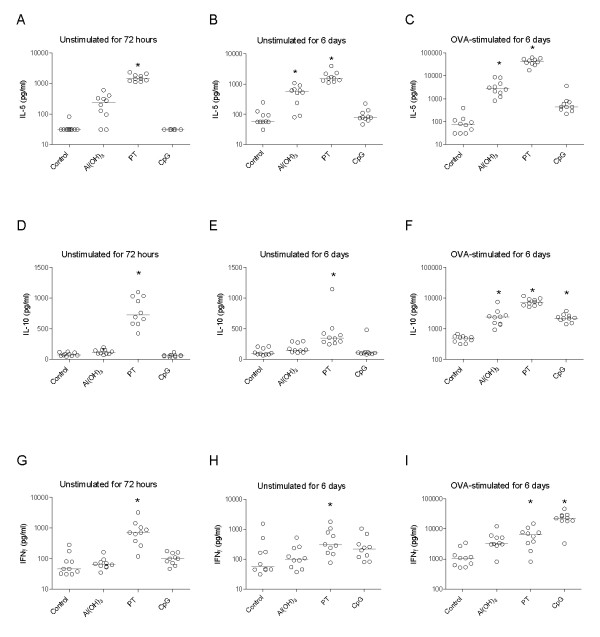
**Cytokine response in spleen cells from mothers three weeks after delivery**. Effect of immunisation of mother with ovalbumin (OVA) combined with either Al(OH)_3_, pertussis toxin, or CpG on cytokine secretion from spleen cells (at weaning three weeks after birth) incubated without allergen for 72 hours and 6 days or stimulated with OVA for 6 days. Ten mothers per group were included. The concentrations of IL-5 (A, B, C), IL-10 (D, E, F) and IFNγ (G, H, I) are given in pg/ml and the median values are indicated by a line, * denotes a statistically significant difference compared to the OVA group, p < 0.05.

Spleen cells from PT treated mothers secreted significantly higher levels of IL-5, IL-10, and IFNγ compared to naive mothers, both in non-stimulated and OVA-stimulated cultures (Figure 2). The cytokine response patterns and the level of cytokines measured in non-stimulated spleen cells after 72 hours and after 6 days were similar. OVA-stimulated spleen cells from Al(OH)_3_-treated mothers secreted significantly higher amounts of IL-5 and IL-10 than cells from naive mothers (Figure 2C and 2F), whereas OVA-stimulated cells from CpG-treated mothers secreted higher amounts of IL-10 and IFNγ compared to naive mothers (Figure 2F and 2I).

### Antibody levels in naive offspring

**Figure 3:**
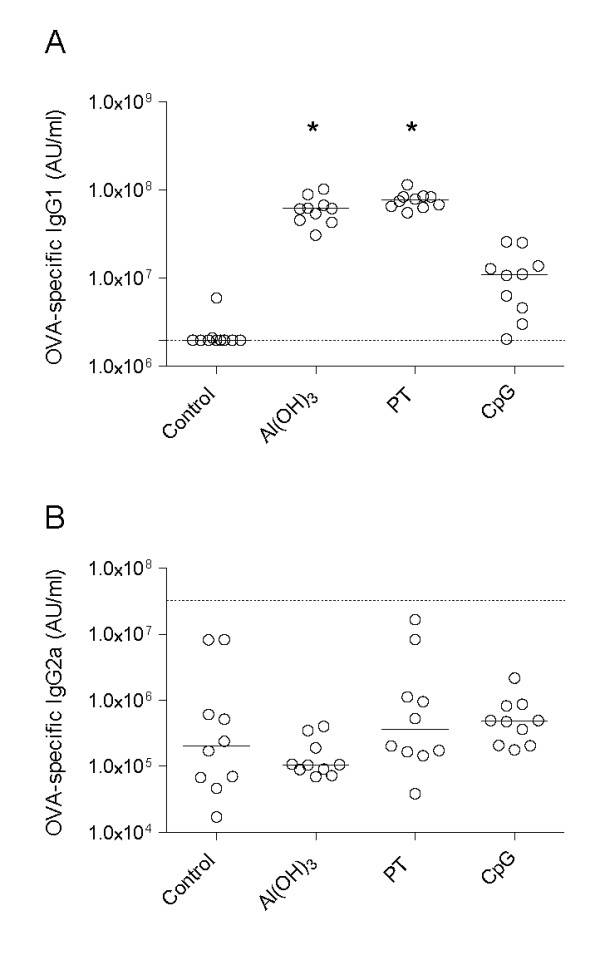
**Antibody responses in non-immunised offspring at 6 weeks**. The figure shows the antibody responses in offspring before the start of immunisation. During pregnancy their mothers' immune response had been polarised towards either a Th1 response ((OVA) + CpG) or a Th2 response (OVA + Al(OH)_3 _or OVA + PT). The antibody concentration for IgG1 (A) and IgG2a (B) are provided in arbitrary units (AU/ml) and the median values are indicated by a line. Forty offspring per group of mother were included; the circles represent the average value of 4 offspring from the same mother, * denotes a statistically significant difference compared to the OVA group, p < 0.05.

Blood samples were taken from offspring before immunisation at six weeks. Higher levels of anti-OVA IgG1 antibodies were observed in non-immunised offspring from Al(OH)_3 _and PT mothers compared to non-immunised offspring from naive mothers (Figure 3A). Similarly, anti-OVA IgG1 antibody levels in non-immunised offspring from CpG mothers tended to be increased (n.s, Figure 3A). No significant differences in anti-OVA IgG2a antibody levels were observed between the groups (Figure 3B). Anti-OVA IgE antibodies were not detectable in any group at 6 weeks in non-immunised offspring.

### Antibody and cytokine levels in offspring at 9 weeks

**Figure 4:**
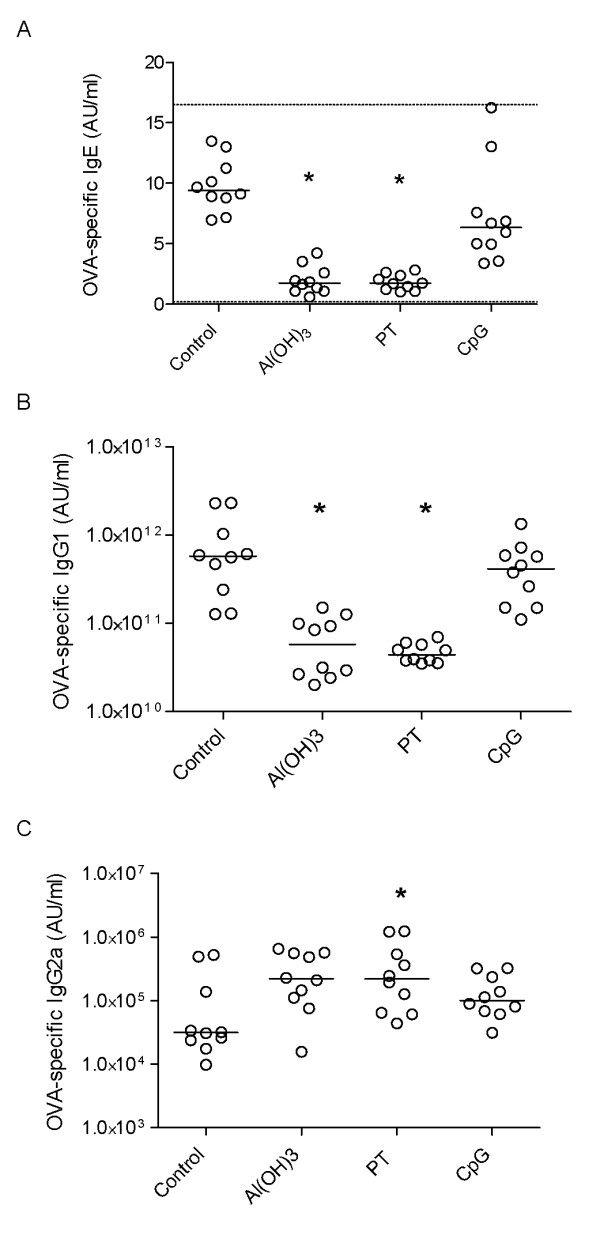
**Antibody responses in 9 week-old immunised offspring**. Antibody responses in allergen immunised offspring after polarisation of the mothers immune response towards either a Th1 response ((OVA) + CpG) or a Th2 response (OVA + Al(OH)_3 _or OVA + PT) during pregnancy. Serum levels of OVA-specific IgE (A), IgG1 (B), and IgG2a (C) antibody concentrations are provided in arbitrary units (AU/ml). The median values are indicated by a line. Forty offspring per group of mother were included; the circles represent the average value of 4 offspring from the same mother, * denotes a statistically significant difference compared to the OVA group, p < 0.05.

To explore possible effects of different adjuvants used for immunisation of the mothers during pregnancy on offspring sensitisation, we immunised offspring with OVA together with Al(OH)_3_. Immunised offspring from Al(OH)_3_- and PT-treated mothers had significantly reduced levels of OVA-specific IgE and IgG1 and increased levels of OVA-specific IgG2a compared to immunised offspring from naive mothers (Figure 4A, B, and 4C). No significant differences in antibody levels were observed in immunised offspring from CpG-treated mothers compared to immunised offspring from naive mothers.

**Figure 5:**
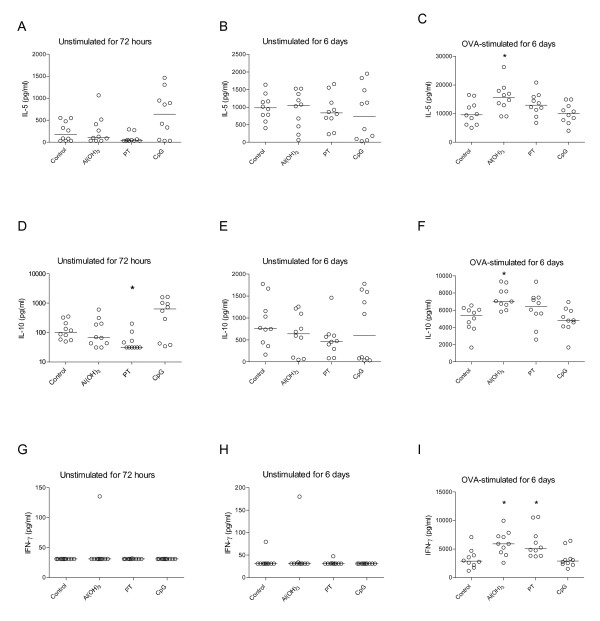
**Secretion of cytokines from spleen cells from 9 week-old immunised offspring**. Cytokine response in offspring from mothers immunised with ovalbumin (OVA) combined with either Al(OH)_3_, pertussis toxin, or CpG. Spleen cells were incubated alone or in combination with the allergen OVA and cytokine secretion were measured after 72 hours (unstimulated) and 6 days (unstimulated or stimulated with OVA). Ten offspring per group of mother were included. The concentrations of IL-5 (A, B, C), IL-10 (D, E, F) and IFNγ (G, H, I) are given in pg/ml and the median values are indicated by a line, * denotes a statistically significant difference compared to the OVA group, p < 0.05.

Spleen cells from immunised offspring of Al(OH)_3_- and PT-treated mothers secreted significantly higher levels of IFNγ after stimulation with OVA compared to immunised offspring from naive mothers (Figure 5I). Additionally, OVA-simulated cells from immunised offspring from Al(OH)_3_-treated mothers had significantly higher levels of IL-5 and IL-10 compared to immunised offspring from naive mothers (Figure 5C and 5F). No significant difference was observed in cytokines levels (IFNγ, IL-10, and IL-5) between immunised offspring from CpG mothers compared to immunised offspring from naive mothers (Figure 5).

## Discussion

In the present study, we confirm and extend previous findings [8] that the capacity for allergic sensitisation to an allergen in young adult offspring can be down-regulated by immunisation of mothers with the corresponding allergen during pregnancy.

By using different microbial components, we aimed at skewing the immune response in mothers towards a Th1 and a Th2 immune response. Increased levels of Th2-associated anti-OVA IgE and IgG1 antibodies were found in PT mothers, whereas CpG mothers had higher levels of Th1-associated anti-OVA IgG2a antibodies. In general, spleen cells from PT mothers secreted increased levels of all three cytokines measured (IL-5, IL-10, IFNγ), and it was not possible to determine a clear Th1/Th2 pattern at the cytokine level. CpG mothers secreted increased levels of IL-10 and IFNγ. As anticipated, also the known Th2-adjuvant Al(OH)_3 _induced significantly higher levels of IgE and IgG1 suggesting a Th2 dominance in the mothers. Taken together, the antibody and cytokine data indicate that CpG immunised mothers achieved predominantly a Th1 immune response, whereas PT and Al(OH)_3 _mothers displayed a Th2-dominated response as intended. However, the distinction was more clear at the antibody than at the cytokine level.

The strongest protection against allergic sensitisation in offspring was found to be associated with a Th2-type immune response in the mothers, rather than with a Th1-type immune response. This finding was demonstrated in two independent experiments (data from one experiment is shown). Two different Th2-adjuvants, the microbial compound PT and the mineral adjuvant Al(OH)_3_, induced Th2-biased immune responses in the mothers and strong reduction of OVA-specific IgE and IgG1 responses in the offspring. The Th1-adjuvant, CpG, gave only slight (not statistically significant) modulation of offspring immune responses with respect to OVA-specific IgE levels. This finding obviously is important in the perspective of adjuvant selection for modulation of allergy development in similar models, which was the purpose of the study. It may also be relevant for the discussion of environmental factors ('natural adjuvants') and allergy development, including the "hygiene hypothesis" [17,18]. However, we have only used one Th1 adjuvant in our study and this limits the generalization of our findings to all Th1 adjuvants.

With regard to communication between the immune systems of mother and child, specific maternal IgG1 antibodies are known to be transferred through the placenta and with milk and have been suggested to be involved in the protective effect on allergy development [19-21]. A protective effect by IgG antibodies may be explained by binding of IgG to the Fcγ RIIb receptor on neonatal B cells, and subsequent cross-linking of the Fcγ RIIb and B cell receptors by antigen, which may lead to inhibition of B cell responses. However, maternal specific IgG may also mask antigenic determinants, and IgG coated antigen may be cleared and destroyed by phagocytosis. Since we found up-regulation of OVA-specific IgG2a levels in offspring, there is no general inhibition of B-cell responses or masking of OVA-antigens in our model. In a study by Seeger *et al. *suppression of IgE against PLA_2 _was efficient also after maternal injection of monoclonal anti-PLA_2 _IgG1 antibodies [22]. The authors argued that it was not likely that one monoclonal anti-PLA_2 _IgG1 antibody could cover the whole range of antigenic determinants and that masking of determinants therefore hardly could be the mechanism for the IgE suppression.

A higher level of anti-OVA IgG1 was found in non-immunised offspring from Al(OH)_3 _and PT mothers at 6 weeks compared to non-immunised offspring from naive mothers. Anti-OVA IgG1 antibodies measured in non-immunised offspring most likely represent maternal OVA-specific IgG1 antibodies transferred transplacentally and via milk. Previously, supposedly maternal IgG1 antibodies have been found in up to 12 week-old offspring [21]. However, theoretically, the antibodies in non-immunised offspring could also be produced by the offspring as a result of maternal influence transplacentally and/or via milk. It has been observed even in humans that maternally transferred IgG to inhalant allergens in cord blood was associated with a reduction of allergen specific IgE and reduced development of later atopic disease in the child [23]. However, it must be remembered that the presence of allergen-specific IgG1 in mouse offspring does not necessarily mean that the IgG1 is the mediator of protection against sensitisation. Even if IgG1 transferred from mother should be the primary "communication" mechanism from mother to offspring, persistence of maternal IgG1 may be irrelevant in relation to protection against sensitisation. The increased levels of IgG2a in offspring of immunized mothers may, hypothetically, have been caused by fetal priming for this isotype, with a concomitant "suppressive priming" with regard to IgE and IgG1 responses. If this is the case, a next question will be the role of cytokines e.g. IL-10 in this hypothetical process of isotype selective positive and negative fetal priming.

Surprisingly, we observed a better protective effect against sensitisation in offspring from mothers with Th2 immunity compared to Th1 immunity during pregnancy. This is in apparent contradiction to the common perception that a Th1-stimulating environment will prevent allergy development [24]. Protection against allergy in offspring has also been reported after injection of mothers with the Th1 cytokine IFNγ during pregnancy [25]. However, our data suggest that IFNγ is not crucial in our model, since spleen cells from the CpG mothers had increased secretion of IFNγ after OVA stimulation, while offspring showed little reduction of IgE production. Although we skewed the antibody response towards either a Th1 or a Th2 response in mothers, we cannot exclude that the effect in the offspring of maternal allergen immunisation may be mediated by increased Treg activity. This could explain the discrepancy between the results found in our study and the results of Matson *et al. *(2007), who observed that Th1 immunity during pregnancy (induced by CFA containing killed *Mycobacterium tuberculosis*) suppressed allergic sensitisation in the offspring more effectively than an Al(OH)_3_-induced Th2 immunity during pregnancy [26]. Killed mycobacteria have been shown to induce regulatory T cells [27]. Some studies have shown a reduced Th2 response in the offspring after their mothers have been immunised with a Th2 adjuvant [28,29], whereas others have not [26,30]. The diverging results could be explained by different experimental designs [26], since Matson *et al. *found protective effects when using Al(OH)_3 _in one of the two studies published using Al(OH)_3 _[26,31]. A slightly different immunisation protocol and dose was used in these two studies [26,31]. This illustrates that the experimental design of the study is of importance. Also timing and route of exposure may be of relevance for a successful protection. Furthermore, other studies have shown an increased Th2 response in the offspring born from allergic mothers [32]. Another problem with comparing these studies directly is the use of different mouse strains, however the protection observed in offspring in our model has been demonstrated in different mouse strains (unpublished data).

The protective effect observed in the offspring after immunising the mothers during pregnancy is very interesting and promising for future prevention of allergy. However, generalisation from mouse to humans can not be done for several reasons, for instance materno-foetal/offspring transmission of IgG in humans and the mouse is different, and human and mouse IgG subclasses do not correspond with regard designations and function.

## Conclusion

Different microbial adjuvants used for maternal immunisation were found to differently affect sensitisation to the same allergen in offspring. The two adjuvants conferring a maternal Th2-type immune response were associated with a stronger reduction of allergen-specific IgE production in the offspring than the microbial component giving a maternal Th1-type immune response to the allergen. However, we do not know whether this is a general principle, or whether it is dependent on the specific adjuvants and/or route of exposure employed. Whether the effect is long-lasting and antigen specific can not be answered by this study. However, the finding that maternal immunisation can suppress sensitisation in the offspring is robust, and the concept of allergy prevention by maternal immunisation should be further explored.

## Abbreviations

OVA: Ovalbumin; PT: pertussis toxin; CpG: cytosine-guanine dinucleotides, Al(OH)_3_: aluminium hydroxide.

## Authors' contributions

LKE participated in the design, coordinated and conducted experiments and drafted the manuscript. UCN helped coordinate the study and participated in the design of the study. IM participated in the design and had done all the pilot studies that the model was based on. ML established the maternal immunisation model and developed the project idea, obtained funding, and participated in the study design. All the authors have critically read, commented and approved the final manuscript.
